# Steroid-induced hyperglycaemia in primary care

**DOI:** 10.1080/17571472.2015.1082344

**Published:** 2015-09-28

**Authors:** Edouard Mills, Senan Devendra

**Affiliations:** ^a^ London Deanery (North West Thames); ^b^ West Hertfordshire Hospitals NHS Trust, Level 3 AAU, Watford General Hospital, Watford WD18 0HB

**Keywords:** Glucocorticoids, steroids, hyperglycaemia, home glucose testing

## Key messages

• Glucocorticoids are commonly used drugs in the inpatient and outpatient settings.
• Their use can cause steroid-induced hyperglycaemia and steroid-induced diabetes.
• High-risk patients for steroid-induced diabetes should undergo blood glucose monitoring and be screened for pre-existing undiagnosed diabetes.
• Sulphonylureas are the mainstays of oral treatment due to rapid onsets of action.
• Failure to respond to oral hypoglycaemic agents suggests that initiation of insulin should be considered.
• There are numerous strategies to promote effective care and avoid hospital admissions safely.

## Why this matters to me

The use of steroids in patients with known diabetes is a common clinical problem, whereby no accepted management strategy exists for when hyperglycaemia results. Much of what is practised is from professional knowledge and clinical experience. Failure to recognise a decompensation in pre-existing diabetes or a diagnosis of steroid-induced diabetes can result in those complications from acute hyperglycaemia. We have aimed to construct a framework for managing hyperglycaemia secondary to steroid therapy, based on a range of consensus documents, professional guidelines and clinical experience. It is written with the intent of helping to improve the care that we give to our diabetic patients by preventing acute diabetic emergencies and avoiding unnecessary hospital admissions.

## Case history

A 68-year-old retired builder (BMI 22 kg/m^2^) who suffers with Type 2 diabetes and Chronic Obstructive Pulmonary Disease (COPD) recently visited his GP due to increased cough and shortness of breath. His most recent HbA1c was 7.3% (DCCT) 56.3 mmol/mol (IFCC). For diabetes control, he was taking Metformin 2.5 grams per day. His GP felt that his symptoms were attributable to a non-infective exacerbation of COPD and prescribed 40 mg of Prednisolone for five days. He had declined to do any regular home blood glucose monitoring. Two days later, he felt more unwell and lethargic. He presented at the Emergency Department and the main test abnormality was a random capillary blood glucose of 21 mmol/l. Urinary testing demonstrated mild ketonuria (1plus on KETOSTIX). He was examined by the diabetes specialist team and discharged after advice and appropriate follow up was given. The case was brought to the Ealing multidisciplinary group meeting to discuss certain issues and whether his admission could have been prevented.

## Ways to think of the problem

### 1. How do glucocorticoids increase blood glucose?

Glucocorticoids are commonly used drugs in the inpatient and outpatient settings for the management of a variety of conditions including inflammatory conditions and haematological malignancies. Their use can result in a number of endocrinopathies and other pathologies, including suppression of the hypothalamic–pituitary–adrenal axis, Cushing’s Syndrome, osteoporosis, weight gain, hypertension and insulin resistance/hyperglycaemia. Hyperglycaemia secondary to glucocorticoids can be seen in those patients with known pre-existing diabetes mellitus, ‘unmasking’ of undiagnosed diabetes mellitus, as well as in a causative manner for the condition (‘Steroid-induced’). The exact prevalence of hyperglycaemia secondary to glucocorticoid therapy is not known, making this an unpredictable challenge for general practitioners and diabetologists alike.

The metabolic effects of glucocorticoids on glucose metabolism are seen at numerous stages in the insulin-signalling cascade. Increased hepatic glucose production occurs via stimulation of gluconeogenesis and glycogenolysis. Inhibition of glucose uptake by muscle and fat, and decreased insulin production and secretion, further promote hyperglycaemia. The typical profile is a pronounced post-prandial glucose rise, as opposed to exaggeration in fasting glucose levels, which peaks at 8–12 h after glucocorticoid intake.

The adverse outcomes of hyperglycaemia have been well documented. These include immunosuppression leading to infections, dehydration predisposing to diabetic emergencies (hyperosmolar hyperglycaemic state, diabetic ketoacidosis or decompensated diabetes), as well as increased risk of all in-patient complications, length of stay and mortality.

### 2. When is it necessary to ask patients to monitor their blood glucose at home?

The Joint British Diabetes Societies (JBDS) recommend that all patients with known Type 1 or Type 2 Diabetes receiving glucocorticoid therapy should monitor their blood glucose by using capillary blood (finger-prick) testing. They recommend that irrespective of background diabetes control, testing up to four times daily and aiming for target blood glucose level of 6–10 mmol/l, although accepting 4–12 mmol/l. In those patients at significant risk of hypoglycaemia, such as the elderly, an individualised and higher target range may be appropriate. [1]

Blood glucose monitoring should also be considered for non-diabetic patients who are at high risk of developing steroid-induced diabetes (Table 1). Where possible, high-risk patients should also be screened for pre-existing undiagnosed diabetes with a fasting-blood glucose and glycosylated haemoglobin (HbA1c) prior to commencing glucocorticoid treatment. In those patients where HbA1c could be unreliable, such as those with haemoglobinopathies, renal failure and anaemia/recent blood transfusion, fructosamine measurement would be a better alternative.

**Table 1.** High-risk patients for steroid-induced diabetes.

• Elderly • Overweight/obese • Strong family history of Type 2 diabetes • Personal history of gestational diabetes or previous steroid-induced hyperglycaemia • Physical examination findings suggestive of insulin resistance, such as acanthosis nigricans) • High-dose glucocorticoids (Prednisolone > 20 mg, hydrocortisone > 50 mg, dexamethasone > 4 mg).

No consensus exists for the optimal screening frequency. We recommend screening with a once to twice weekly 2-h post-lunch capillary blood glucose in those patients who are not known to be diabetic, but at high risk of steroid-induced diabetes. A capillary blood glucose of ≥11.1 mmol/l needs to be repeated as a random venous sample to confirm a diagnosis of steroid-induced diabetes. There is no place for the use of HbA1c in diagnosing steroid-induced diabetes.

### 3. What are the current regimens available for managing people with hyperglycaemia who take glucocorticoids?

At present, there is no consensus guideline for the optimum management of hyperglycaemia secondary to glucocorticoids, although varying international opinions exist. The JBDS for Inpatient Care group published recommendations in October 2014 for the management of hyperglycaemia and steroid therapy. General international recommendations, including from the Canadian Diabetes Association and Australian Diabetes Society, support the use of insulin. [2, 3] We believe that the factors which should influence the decision of when and how to treat should include the intended duration of glucocorticoid therapy (especially if greater than four weeks), the frequency of glucocorticoid doses, the severity of hyperglycaemia, the underlying condition and commodities, as well as patient choice.

#### Oral hypoglycaemic agents

Although all oral agents can be theoretically used to treat steroid-induced or steroid-exacerbated diabetes, preference should be given to those agents which target post-prandial hyperglycaemia and have a rapid onset of action. Prescribers must recognise that certain oral hypoglycaemic agents are contraindicated in the context of renal, hepatic or heart failure. Initiation of treatment should be considered once home blood glucose monitoring yields persistently high readings of 9–15 mmol/l.

We would recommend considering:

• Sulphonylureas

Second-generation sulphonylureas, such as gliclazide and glibenclamide, are the mainstays of oral treatment due to rapid onsets of action. Doses should be up-titrated every 48–72 h to gliclazide 160–240 mg/day or glibenclamide 2–3 mg/day, without causing significant risk of hypoglycaemia. Doses are most appropriately given at lunch time to target post-prandial hyperglycaemia. Should hyperglycaemia remain an issue at near-maximum dose, initiation of insulin should be contemplated rather than the addition of an alternative oral hypoglycaemic agent as these act too slowly to be beneficial in this circumstance. At this point, it would be appropriate to discuss with a specialist diabetes team.

• Meglitinide analogues (Prandial glucose regulators)

Repaglinide and nateglinide have a much more rapid onset and offset of action than the typical sulphonylureas, thereby reducing the risk of hypoglycaemia. By taking them immediately before a meal, they are able to control the post-prandial hyperglycaemia seen with glucocorticoids within less than one hour, with a length of action of 4–6 h. Disadvantage is the requirement for multiple daily doses.

• Subtype 2 sodium-glucose transport protein inhibitors (SGLT-2 inhibitors)

These novel once daily agents include dapagliflozin and canagliflozin. They function by reducing glucose resorption at the kidneys, thereby promoting glucose excretion in urine. Canagliflozin, for example, is rapidly absorbed in the gastrointestinal tract and reaches peak concentrations within the systemic circulation at 1–2 h. [4] In our clinical experience, we have observed rapid reduction (within 48 h) in glucose levels upon taking this class of drug.

#### Insulin

The benefits that insulin offer are greater flexibility and predictability, rapid ability to target post-prandial hyperglycaemia, dose modification related to patient oral intake and unlimited dosing. Initiation of insulin does require patient education and support to recognise the clinical features of hypoglycaemia and more stringent monitoring.

The typical starting dose for an insulin naive patient can be calculated at 0.3–0.5 units/kg for a 24 h period (‘estimated total daily dose of insulin’). The regimen of insulin will be dependent on whether both the fasting and post-prandial glucose readings are raised, or only post-prandial as well as the frequency of glucocorticoid administration is increased.

• If only the post-prandial glucose is elevated and administration of glucocorticoid is daily once, preference should be given using basal analogue insulin such as Human Insulatard, Insuman Basal or Humulin I and dosing around the time of the glucocorticoid dose as this will parallel the glucose rise. We favour this approach initially as it potentially avoids the need for multiple daily insulin doses. Rapid acting insulin analogues with lunch and evening meals can be added if fasting glucose remains controlled at <7 mmol/l, but other readings are persistently high.
• In those patients requiring multiple daily doses of glucocorticoids, an intermediate acting insulin with meal-time rapid acting analogues could be initiated. The proportions of the estimated total daily dose of insulin would be 30% basal and 70% bolus divided across the meals.

#### Pre-existing diabetes

Those patients who are diet controlled may require an oral hypoglycaemic agent, similar to patients at high risk of developing steroid-induced diabetes.

Those patients who are already on an oral hypoglycaemic agent may require dose intensification, addition of an alternative agent (preference given to sulphonylureas and up-titrating the dose to maximum) or initiation of insulin if despite these measures, blood glucose levels persistently >10 mmol/l. On starting insulin, the dose of sulphonylureas should be reduced to prevent hypoglycaemia, whilst other oral hypoglycaemic agents can be continued.

Those patients who already require insulin will require dose titration to maintain appropriate glycaemic control, often in 20% increments of daily dose. Re-evaluation will need to take place daily as the effect of glucocorticoids on glycaemic control is cumulative. In those patients on a basal-bolus regimen (four to five doses per day), it may be necessary to change the ratio from 50% basal/50% bolus to 30% basal/ 70% bolus, or keeping the same basal dose and increasing the meal insulin. In those patients only on a basal insulin with suboptimal glycaemic control, the addition of rapid acting insulin analogues with lunch and evening meals may be required.

### 4. How can we avoid possible admissions in people with hyperglycaemia who take high-dose glucocorticoids?

• Ensure all diabetic patients and high-risk patients for steroid-induced diabetes have access to blood glucose monitoring, to prevent the development of hyperglycaemic emergencies. [5]
• All patients should be educated on typical hyperglycaemic symptoms, which should prompt them to check their capillary blood glucose.
• A care plan for patients with strategies to intercept and manage hyperglycaemic and hypoglycaemic episodes, would be particularly useful out-of-hours.
• Once the dose of glucocorticoids are being tapered down, the dose of oral hypoglycaemic agents or insulin should be appropriately reduced to prevent hypoglycaemia.
• GP access to quick advice (over the phone or by e-mail) to structured diabetes clinics led by general practitioners with an interest in diabetes, diabetes specialist nurses, nurse consultants and secondary care input.
• Appropriate follow-up of previously unknown diabetic patients with an HbA1c 12 weeks following completion of glucocorticoid therapy to re-assess diabetes status.

## Outcome of the case

Once our patient was started on Gliclazide 80 mg at lunch time, within 48 h his post-lunch capillary blood glucose reduced from 18–12 mmol/l. Two days after stopping prednisolone, his post-prandial lunch glucose levels reduced to 9 mmol/l. Subsequently his Gliclazide dose was stopped. If he does require glucocorticoid therapy in the future, he will be asked to monitor his capillary blood glucose at home and should have access to timely intervention with appropriate glucose lowering therapy.

## Ethics Approval

Not required
